# University of Pennsylvania - Dental Department

**Published:** 1882-05

**Authors:** 


### UNIVERSITY OF PENNSYLVANIA—DENTAL DEPARTMENT.

Third annual commencement.

The number of matriculates for the session was eighty-eight.

The degree of D.D.S. was conferred on the following members of the class by William Pepper, M.D., Provost of the University:

| NAME. | RESIDENCE. | NAME. | RESIDENCE. |
|---|---|---|---|
| James H. Abrams | Pennsylvania. | John H. Laney | Missouri. |
| Edward H. Allen | Illinois. | Martin H. Musser | Pennsylvania. |
| Philip Betts | England. | A. W. McCandless | Iowa. |
| Allen E. Bradley | New York. | Harry B. McFadden | Pennsylvania. |
| Albert J. Bushong | Pennsylvania. | Charles R. McFarlan | Pennsylvania. |
| Chas. O. deCausse, M.D. | Cuba. | Frank H. McIntire | Massachusetts. |
| Alfred E. Correvan | Switzerland. | William McNair | Pennsylvania. |
| Alfredo Cristy | Porto Rico. | Leoncio B. Nunez | Cuba. |
| Theodore E. Devereux | Wisconsin. | Gabriel Oltramare | Switzerland. |
| Raphael A. C. Dillon | Brazil. | Chas. J. Peters, Jr | New York. |
| J. Judson Edwards | Pennsylvania. | Manuel G. Ramos | Ecuador. |
| Adriel B. Ely | New York. | Godfrey S. Salomon | Wisconsin. |
| Francisco Escobar | U. S. of Col. | Wm. H. Shannon | Pennsylvania. |
| J. A. Fothergill, L.D.S. (M.R.C.S.) | England. | John G. Sharpe | New York. |
| Solomon Freeman | Pennsylvania. | H. L. Smedley (Ph. G.) | Pennsylvania, |
| William E. Gerrish | Indiana. | Charles M. Stetson | Buenos Ayres. |
| Emil Haberstich | Switzerland. | Eugene Sunderland | Illinois. |
| George L. Hurd | Massachusetts, | John W. Tudor | Pennsylvania. |
| Henry H. Keim | Pennsylvania. | Albert G. Weed, Jr | Connecticut. |
| Charles F. Kelly | Pennsylvania. | William T. White | New Jersey. |
| | | Jefferson P. Winner | Delaware. |

